# The Educational Function of English Children’s Movies From the Perspective of Multiculturalism Under Deep Learning and Artificial Intelligence

**DOI:** 10.3389/fpsyg.2021.759094

**Published:** 2022-01-24

**Authors:** Nan Hu, Shuyi Li, Luna Li, Hui Xu

**Affiliations:** ^1^Foreign Language School, Guangxi Medical University, Nanning, China; ^2^School of Arts and Communication, Beijing Normal University, Beijing, China; ^3^School of Educational Studies, Universiti Sains Malaysia, Penang, Malaysia; ^4^Homerton College, University of Cambridge, Cambridge, United Kingdom

**Keywords:** multiculturalism, positive psychology, English children’s films, educational function, deep learning, artificial intelligence

## Abstract

In children’s learning subjects, English courses has its relative particularity compared with Chinese courses and the mathematics. Children’s English teaching is often inefficient because of the lack of students’ timely consolidation after class. Given this, the present work starts with the analysis of the current situation of children’s learning, and introduces the film-assisted English teaching. In the specific teaching links, English teaching is carried out in a three-dimensional teaching mode. Before that, topics of the films are selected for the English teaching, and the films are edited and processed. Initially, the present work expounds the English children’s films and their educational functions. Then, children can obtain teaching effect from the films. An English questionnaire is designed to analyze the application effect of English films in children’s English teaching. The results show that the film teaching mode improves children’s learning interest and motivation, and English film teaching can stimulate students’ learning interest. Students are also more active to participate in teaching activities, thus improving their language skills. Under the teaching in the scenes of films, students can perceive the functions of language in certain contexts. Comprehensible language input promotes students’ English listening ability and oral expression ability. The films can intuitively show the humanistic style, historical geography, cultures, and customs of English countries, and then cultivate students’ western cultural literacy. Practice has proved that the method proposed here can achieve good teaching effect, and it provides certain references for children’s English education.

## Introduction

In China’s education reform program, it is emphasized that the application of information technology and its combination with education must be vigorously promoted for China’s basic education. Modern information technologies provide people with better video resources to reform education methods, update teaching content, and make up for the shortcomings of previous classroom teaching (Wu Y. J. et al., 2020; Yuan and Wu, 2020; Wang Y. et al., 2021). English teaching in primary school helps to improve the basic English ability of children, and stimulate students’ positive attitude toward English learning under the consideration of the current situation and characteristics of the student development in basic education stage (Wu and Wu, 2017; Wu W. et al., 2020). In this way, students will feel easy and confident in learning English. Thereby, education should make students active and positive in learning and thus cultivating their learning initiative. English plays a very crucial role in every stage of learning of Chinese students. Primary school is the key period to lay the foundation of English for a student. The interest and time of English learning in primary school will always affect the students’ English learning in middle school and even college. English is an international language, and every English teacher should try to help students develop interest in English and help them master relevant knowledge and skills. English teaching in primary school should focus on students’ listening, speaking, reading and writing (Zheng et al., 2018; Kliziene et al., 2021). The quality of teaching strategies determines students’ interest in English learning.

The learning and life of the new generation of children are changing with the rapid development of network technology and film production and the rapid emergence of various films. The plots and pictures of children’s films can be used to attract students to learn English. When the film is played, English vocabularies automatically enter the ears of students. Cartoon is one of the kinds of films, which has animated characters with rich colors, fast rhythm and bright colors. Cartoon can arouse students’ enthusiasm and help them learn English (Qian et al., 2018; Kyriakidis et al., 2021). Cartoon can make abstract knowledge easy to understand. Teachers should let students explore and study actively, and more importantly, let students acquire knowledge in happiness (Hayashi et al., 2016; Alharbi, 2021). These unique characteristics of film are in line with the needs and development of students. Film can maximize the effect of teaching to stimulate students’ interest and motivation, and also can make students study actively in a relaxing atmosphere, so that children make themselves as the master of the class, which realizes the auxiliary role of cartoons in English teaching (Rogoza et al., 2018; Chen, 2019). The plots, contents and pictures of the cartoon attract students to learn English. Besides, children will automatically be familiar with English vocabularies when they watch cartoons (Wu et al., 2019; Wu and Song, 2019). English teaching with films can fully use the characteristics of films to stimulate students’ interest and enthusiasm in learning, and also enable students to actively learn and explore in a pleasant atmosphere (Wang S. et al., 2021; Yulia and Amirudin, 2021). The reason is that the vivid pictures, dynamic music and vivid characters relieve children from the boreness and tediousness of learning, which improves their effect of English teaching. In this way, children can spontaneously achieve the learning goal in the classroom, which also realizes the role of films in assisting English teaching (Takahashi and Sato, 2020; Breda et al., 2021).

The study is conducted on the application of English children’s movies in their language education. By investigating the relationship between children’s learning style and learning retention rate, it is found that children can better memorize the information obtained through visual, acoustical, and other channels. Besides, when vision is combined with hearing, there will be higher rate that children receive massages. Therefore, English teaching should make the best of audio-visual resources and other materials to improve children’s learning and memory ability. The above research results lay the foundation for the follow-up study in present work. Present work then designs the teaching mode of children’s animation, selects “*Snow White*,” “*Croyz*,” “*Lion King*” and “*Witch with Black and White Hair*” four films as teaching materials, and combines the course to study the teaching of students. A questionnaire survey is conducted on students after teaching. Some survey results show that with the continuous growth of film teaching time, students’ mastery of knowledge is better. Most students like film teaching. They think film teaching helps to understand western culture and makes them more interested in English.

The purpose of present work is to change the current situation of children’s English learning and solve the problem of low efficiency caused by the lack of timely consolidation after English teaching. The proposed solution is to construct a comprehensive English teaching mode of “seeing, listening, speaking, reading and writing” through the introduction of English movies, to achieve good teaching results. The limitation of the method is that it is difficult to completely collect relevant movies or movie clips that match each knowledgement unit of the textbook, so the film teaching method is carried out only in some them effectively. Through the study, it is expected to enable children to learn more English vocabulary and sentences and cultivate their interest in English through the proposed method. The beneficiaries of present work are preschool children. Based on this, with primary school students as the research object, the questionnaire survey is carried out to analyze and summarize the function of the film in teaching activities. The innovation is to apply film teaching in English teaching of primary school based on the rapid development of modern internet technology support. This exploration provides some reference value for the implementation of English films in children’s English teaching.

## Literature Review

In terms of children’s English teaching, what is illustrated are the educational quality of Swedish primary school, and views on the prospective system of children’s personalized development of reading skills in English classroom. The project aims to develop children’s personalized reading and learning skills. Analyzation is made on the semi-structured interviews with 11 primary school teachers to solve the actual problems. The analysis shows that Swedish teachers have great differences in cultivating the children’s English ability with Chinese teachers. The former believes that personalized learning skill is a promising method to alleviate the differences of the children’s learning ability (Bunting et al., 2020). Theoretical and empirical analyses are conducted on the problems existing in primary school English teaching. Teachers should form a positive method to demonstrate students’ knowledge and ability. Thinking in a foreign way is set as the goal of children’s learning and the indicator of the proficiency of a foreign language. Attemption is made to evaluate the heuristic and developmental resources of metacognitive strategies in the logic of projective recursion for English teaching in primary school. A research result is proposed on primary school students’ dominant thinking type as an indicative construct of metacognitive development. Research shows that most of the junior students have the characteristics of symbol and the ability of symbolic thinking, which confirms the possibility of using metacognitive programs in English teaching for children (Tokareva and Tsehelska, 2020). In terms of children’s teaching and multiculturalism, it is pointed out that multicultural curricula poses several challenges to educators. What is introduced are the basic principles of using relationship teaching method to teach multicultural courses, including three classroom activity culture theories based on relationship teaching method and relationship teaching method. By using the relational approaches, teachers can encourage students to focus more attention in learning inclusiveness, competence and cultural awareness consultation practice, and make them more self-motivated and more willing to put forward questions (Cort et al., 2020). Discussion is made on the implementation of multi-disciplinary and multi-cultural student team cooperation, case-based learning and problem-based learning as a prospect of sustainable teaching practice. Based on the mixed method, including direct observation (physical and virtual), questionnaire and learning focuses group, research shows that this method can mostly promote but also can lightly hinder the sustainable learning (Doukanari et al., 2021).

## An Analysis of English Children’s Films and Their Educational Functions

### The Relationship Between Deep Learning and Children’s Education

**FIGURE 1:**
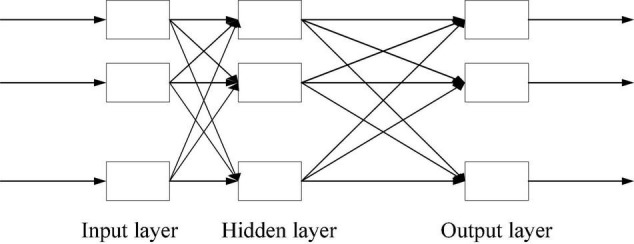
Three-layer model of DL.

The application of artificial intelligence (AI) in the field of education is triggering a new revolution. From automatic teaching to the development of automatic response evaluation system, from the decision-making based on single-mode data to the multi-mode supporting data, the inherent power of AI is gradually infiltrating into classroom teaching. In the whole process of education, the ability-based teaching method is the requirement of educational fair value. The deep integration of AI and education has resulted in a fundamental change in the form of education, and revised the relationship between teachers and students, which can help learners adapt and learn individualizedly, and provide a new way to achieve educational equity (Stadlinger et al., 2021; Zhou et al., 2021). Deep learning (DL) is an important technical means to achieve AI, which is considered to be one of the key technologies to promote the explosive growth of AI research and application. At present, AI products based on DL have been widely used in smart home, intelligent logistics management, intelligent control and other fields. In the field of education, DL plays an irreplaceable role in exploring learners’ learning situations, obtaining learners’ real needs, and diagnosing learners’ weak learning connections. DL is a method of learning data-based representation in automatic learning. Analytic learning is carried out through neural network simulating human brain. Figure 1 displays the three-layer model of DL. In addition to the input layer and output layer, DL also has a powerful hidden layer, which can combine the characteristics of hidden layer to analyze data layer by layer.

### Positive Psychology and Film Teaching Theory

Over the past 5,000 years, Chinese civilization has been developing continuously, and the unity and pluralism of the Chinese nation is the result of historical development. National identity and the history of national culture have been widely studied. The cultural exchanges among people of all countries have gradually produced different characteristics between nationalities and cultures after the founding of the people’s Republic of China (Stogianni et al., 2021; Virgona and Kashima, 2021). The main culture of ethnic groups lies in that different minorities care for each other, interact and infiltrate each other, leading to the coexistence of multi-ethnic cultures in a multi-ethnic country, and representing a multi-ethnic, multi-cultural and multi-community country, thus forming a set of cultural “diversity” (Mekli et al., 2016; White et al., 2016). The uniqueness and variety of the Chinese nation is an inevitable fact of historical development, which makes pluralistic and integrated education an inevitable trend of ethnic education in the future. Therefore, multicultural education is a crucial basis to study the teaching effectiveness of teachers in ethnic education.

Positive psychology is a subject concerned with human well-being and health. As a supplement to psychology, positive psychology focuses less on people’s positivity. It excavates people’s positive potential and returns to people’s rationality. Psychology traditionally focuses on the *status quo* of negative psychology and pathological psychology, while positive psychology embodies the spirit of the times and makes people complete themselves and truly happy (Smith et al., 2019; Zhao and Yang, 2021). The application of positive psychology theory in subject teaching is not very extensive, but there are some attempts about the application of its basic theory in subject teaching. A breakthrough about the basic theory of positive psychology is made, and the teaching design and learning effect are enhanced, which can make children grow more robust and healthy.

TV series, movies, film, and variety shows all belong to the combination of film industry and sound products. Film is the general name of film works. The mentioned film teaching is mainly based on English film to achieve the purpose of teaching, and the teaching mode focuses on the students’ comprehensive training. Film teaching can also be called audio-visual teaching. Liu held the opinion that “Audio-visual teaching is a teaching method that combines images and recordings in certain situations.” Film teaching provides an audio-visual feast for students, and also has gratifying significance (Cherrie et al., 2021; Yth et al., 2021). Film teaching has attracted many scholars with its unique charm. From a certain perspective, English film teaching is conducive to the mastery and learning of English knowledge, as well as the improvement of English expressing and listening ability of students. Wu et al. (2018) analyzed education and proposed to use information technology for analysis and research, which strongly confirmed that science and technology are very feasible in education (Wu et al., 2018).

**FIGURE 2:**
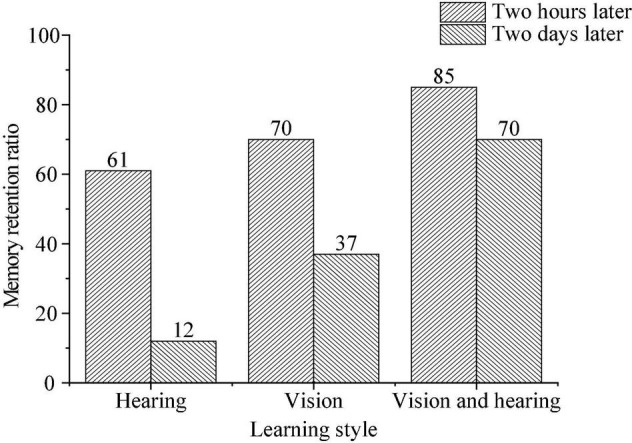
Comparison of learning style and memory retention ratio (data comes from accenture: https://www.accenture.com/cn-zh).

From the perspective of memory, different teaching modes have different retention rates of the learned knowledge, as shown in Figure 2.

Figure 2 signifies that information can be better remembered if it is acquired through multiple channels, such as vision, hearing and kinesthetics. Second language teaching should make full use of materials such as audio-visual resources to improve learning and memory ability of children.

**FIGURE 3:**
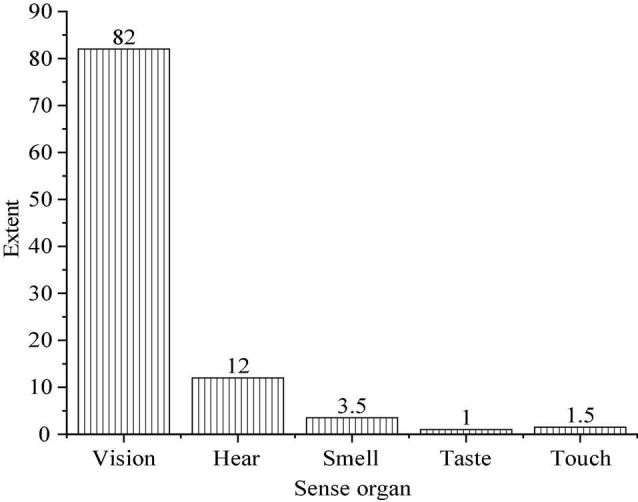
The information acceptance degree of different senses (data comes from accenture: https://www.accenture.com/cn-zh).

Psychology implies that there are different degrees of acceptance and different times of obtaining information when people use different sensory organs to understand and obtain things, as shown in Figure 3.

Figure 3 reveals that people receive 82, 1, 12, 1.5, and 3.5 pieces of information through vision, taste, hearing, touch and smell, respectively. The information reception rate will be significantly improved when vision and hearing are combined.

### Analysis of Learners’ Characteristics

Compared with the lower grade students, the selection of students about 11 years old are more efficient, since they have a certain ability of expression, and are willing to express their personal views, explore new knowledge, and actively participate in activities. However, primary school students’ attention is easily interfered by the outside world, and they are unable to concentrate on long-term stable learning. The students of this level have not developed their logical thinking ability, so they are more inclined to memorize images and things that have practical significance and may be of interest. They are not capable to think the more logical content of abstract things. Thereby, creating a suitable real learning environment is of great significance to improve students’ memory. Zheng et al. (2020) emphasized that a certain environment is needed for children education. Regarding learning style, students have low cognitive level, less knowledge reserve and poor logical thinking ability (Zheng et al., 2020). Regarded learning motivation, compared with other age groups, students of this level have little pressure to enter higher education; their main motivation for learning comes from the encouragement of teachers and parents or through the competition among their classmates. The lack of these external factors will exert a great impact on students at this level (Wang S. et al., 2021). Hence, students must be guided to be interested in English subjects spontaneously. The communication with student reveals that students do not like teachers’ simple guidance and recitation, so the classroom atmosphere is not active and students’ overall motivation is not strong.

### The Role and Significance of English Films and Deep Learning in Children’s English Teaching

#### Children Are More Attracted by the Visual Impacts

At present, most schools are equipped with multimedia equipment, which can be used to play cartoons directly and can save time. Before playing an films, it’s best for the teachers to give a brief introduction of the storyline in the film, and by the way, ask some simple questions, using them to attract children ‘s attention and letting them find the answers to these questions from the film. In fact, when watching an films, children generally do not understand the language of them, but they can understand the story plot, know what they are talking about, which is the most important. After playing the cartoon, teachers can ask children to tell what the cartoon tells first, and then explain the questions raised before. Children will actively express their views, so that the purpose of visual impacts can be achieved.

#### Rehearsal Skill of Children Can Be Improved by Listening

In the process of watching films, the specific images of the pictures bring a strong visual impact to children. Besides, lively and interesting language will also leave a deep impression on students’ listening. As mentioned above, children cannot fully understand the language of an film played only once, and teachers need to play it again. In the next process, some children can retell some English conversations in relevant situations. Although they have not been able to accurately translate English into Chinese, children will also remember these English sentences without the need for teachers to punctuate on them. When the teacher plays the films again, children can follow the cartoon to read the sentences under teacher’s instructions. This time, teachers must encourage children and allow some children to read in public, affirming their progress while pointing out some of the small problems. It is important to note that although English cartoons are aimed at children, there are also many English idioms and Westerners’ ways of thinking in English cartoons. Some dialogues are difficult to understand and need to be understood by students through Chinese and English subtitles. When children begin to study, they can understand English with the help of Chinese thinking mode, while thinking and reciting. After a period of time, they should consider directly using English thinking mode to understand the film.

#### English Vocabularies of Children Should Be Expanded

Some schools with good conditions have begun to invite foreign teachers to teach oral English. It is very useful for students with certain English vocabulary accumulation and basic language knowledge to learn from foreign teachers, because they can learn the most authentic English and quickly improve their oral communication ability in English. But if children’s vocabulary is still less than three or four hundred and with a majority of nouns, foreign teachers cannot play a good role in English teaching. Because the scope of conversation between them and students is too small and the efficiency is too low. However, the advantages of the films is reflected under this scenario. Films provides a combination of voice and image for the expansion of children’s vocabulary, especially those complex and abstract new words. Films tend to express the meaning of these words through specific situations, which is beneficial to children’s understanding and memory of vocabulary, and its effect is often better than the traditional education method of language interpretation. Films will appear with the development of the plot and the change of the situation, which has a positive effect on children’s correct understanding of new words. Children will automatically and consciously carry out delayed imitation to learn of these words, so that these words can be added to their English vocabularies. Having learned new words from films, students can make a choice between the words they learned from textbooks, and some commonly used words in daily life they learned from films.

Compared with other modern technologies, the essence of the application of AI is embodied in the “intelligent,” which is also the fundamental foothold in the field of education and teaching. English teaching activities are a complex process, which also contains many uncertain factors, such as those from language teaching software and hardware environment, English teachers’ professional level, students’ interest state, cognitive style, teaching and learning style. The biggest feature of the AI is to simulate human thinking mode and reproduce the process of thinking and reasoning. Therefore, the complexity of the English teaching process needs to play its unique solution strategy to help specify dynamic teaching strategies suitable for each student. The dilemma of poor teaching quality and effect of children’s English requires the reform of science and technology. The advanced intelligent devices in the field of language learning can complement many shortcomings in children’s English teaching, not only conform to the curriculum standards of children’s English, but also change the teaching mode of children’s English class and improve children’s learning style. Besides, the lack of practical application and communication ability of traditional English teaching has always plagued English teaching in China. The application and popularization of AI English teaching system in children’s English teaching will be a breakthrough in the reform of foreign language teaching and the improvement of foreign language teaching quality by modern information technology. It is the peak of improving computer language assisted teaching by intelligent methods by now. Children English teachers should pay attention to personalized learning, pay attention to cultivating students’ oral ability, and begin to stimulate each child’s interest in language learning. These are exactly the essence of AI English teaching system in children’s English teaching, and also a valuable exploration of the integration of modern information technology with language teaching. The most important, the application field needs to be expanded and practiced in children’s English teaching, because education is the base of social progress, and the application field should be expanded to the field of education and teaching. Language is the intelligence that human beings are most proud of. Whether the research of Turin test, natural language processing, machine translation, speech recognition technology, or other application in the field of language are closely related to language learning. How to make computers understand human language and realize human-computer dialogue has always been the most popular research field of scientists. It is possible to realize the application of AI in English teaching.

With the continuous advancement of economic globalization, the cultural life of Chinese is increasingly showing the trend of multicultural coexistence. A distinctive feature of multicultural coexistence is the diversification of language use. The further deepening of reform and opening up causes that China has made great strides to be converged into the world. Different people in the world and information of different culture are appearing in Chinese society. People need to communicate in multiple languages, especially in a globally common language such as English. Therefore, Chinese need to constantly improve their ability of using English. However, at present, people’s ability of using English is not very well. The phenomenon of “English mute” is serious, the living world lacks the environment of English communication for people, the problem that most adults cannot correctly use English is prominent, and children are also deeply affected by the environment. Based on this grim reality, it is believed that English education must start with children. Therefore, attention should be focused on the correct orientation of preschool children’s English education under the multicultural background to guide children’s English learning. Therefore, the correct positioning of preschool children’s English education should be considered under the multicultural background, and children’s English teaching and Chinese teaching should be guided to promote each other, which is the core to carry out early childhood English education, cultivate English sense and guide perceptual culture. “People’s phonological perception is formed in the pre-school stage, and second language phonological perception is also easier to obtain in this period.” Professor Zhou Jing pointed out. In real scenarios of children’s English teaching, the core goal is to cultivate children’s English language sensitivity. The main task is to let children learn in the English atmosphere, contact with English, perceive English, and obtain good language sense, to establish language sensitivity in English earlier.

Positive psychology emphasizes exploring and studying students’ various explicit and potential positive qualities, and expanding and cultivating the positive qualities found in educational practice. In children’s English teaching, the application of positive psychology can help children cultivate positive emotions and explore their own potential. Simultaneously, it can also promote the establishment of a good teacher-student relationship and improve the effect of classroom, teaching. Positive teaching evaluation for students cultivates students’ positive learning experience. Positive mentality is the training goal of positive psychology, including cognitive quality, emotional quality, will quality, and personality quality. Integrating the concept of positive psychology into children’s English teaching is helpful to develop students’ positive psychological quality, including attention, self-confidence, endurance, adaptability, emotion regulation ability and self-management ability, which requires teachers to pay attention to students’ feelings and experiences in English learning while paying attention to teaching and achievements. Educational psychologists believe that emotion and cognition are two inseparable parts of human spiritual world. Students can achieve better cognitive effect with positive emotional participation, and the improvement of cognitive effect can make students have positive emotional experience. In the process of children’s English teaching, teachers should change the concept of evaluation, infect students with positive emotions, understand students with a positive attitude, find out the characteristics and advantages of each student, put forward the goal to adapt to the ability of students with different foundation, and achieve the goal in stages, affirming every progress of each student, so that students get positive incentives in the evaluation system, and they get positive emotional experience in the process of progress, improve self-confidence, get initiative stimulated, and the goal can be realized of the coordinated development of students’ emotion and cognition.

### The Implementation of Deep Learning in Teaching

**FIGURE 4:**
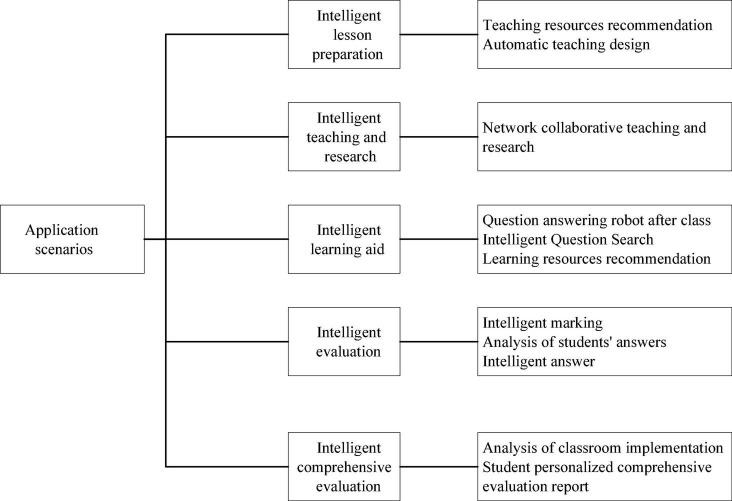
The application of DL in teaching.

In the process of campus construction, relevant enterprises have applied learning monitoring, intelligent tutoring and product examination through DL, and these applications are based on the educational application scenarios of DL. The construction of scenario based smart campus ecosystem can obtain continuous support through DL, and realize the personalized change of regional intelligent education cloud, intelligent campus and intelligent teaching place. Taking intelligent classroom as an example, DL will support the whole process of education and teaching in five application scenarios: intelligent lesson preparation, intelligent teaching and research, intelligent learning assistance, intelligent evaluation and intelligent comprehensive evaluation. Figure 4 illustrates the specific situation of the DL application, which provides students with more intelligent and personalized learning experience.

DL is a learning method which can improve learning ability, practical ability, and innovative ability of students. Students can establish contact with the existing knowledge based on understanding and applying it flexibly to solve practical problems. DL is not passive learning, but active and autonomous learning. It emphasizes that the old and new knowledge are organically linked rather than isolated knowledge fragments; it focuses on meaning of learning, rather than mechanical learning; besides, it also emphasizes the learning method of self-exploration rather than blind acceptance of knowledge. Thereby, DL can mobilize students’ English learning. In order to cultivate learners’ ability of self-exploration and life-long learning, knowledge application ability and innovation ability can be adapted to the training needs of innovative talents in today’s knowledge age. DL is integrated into the specific teaching process. Initially, pre-evaluation, including pre-evaluation of courses and students, is conducted; then, a good learning atmosphere is created to make students keep the best learning state; next, the students’ previous knowledge is stimulated to realize the combination of new and old knowledge; afterward, the teacher guides the students to process the original information to make it their own knowledge and solve problems; finally, students’ learning is evaluated to timely feedback problems, modify learning methods, and promote students’ further learning.

## Questionnaire Design for Teaching

### Experimental Methods and Hypothesis

The experimental purpose is to use movies as supplementary materials for teaching materials, to create real teaching situations for students through multimedia equipment assisted teaching, to stimulate students’ enthusiasm for English learning, to improve students’ English ability in listening, speaking, and reading, and to cultivate students’ English cultural literacy through the use of movies and the design of teaching activities around movies. This experiment adopts the action research method, observation method, questionnaire survey method, and so on. The action research method refers to: first, the investigation and study of the application of English films in children’s English teaching by personally participating in the English film teaching; second, the investigation of the English film teaching for the experimental group students; third, the analysis of the effect of the application of English film teaching. It is adopted to understand the problems in English film teaching. The observation method means analyzing the advantages and disadvantages of English movies in teaching, by observing students’ learning status in the teaching process.

The present work conducts and the following hypotheses and studies on them. They are specifically: (1) The use of film teaching mode in the classroom is appropriate. (2) After the application of film teaching, students have a good grasp of knowledge. (3) Students like film teaching. (4) There are sufficient teaching resources in video teaching. (5) English video appreciation class can improve students’ English level. (6) Students like cartoon-style movies. (7) Students want English classes based on listening.

### Teaching Content Design

Before class, the English teacher can tell students the name of the film to be used. And teacher plans the text and related knowledge points according to the content to be taught. In this case, it is easy to attract children’s attention and stimulate their interest in watching films before class. Although students do not know what to learn, this process can make students effectively pre-understand part of the teaching content in autonomous learning. The memory theory involved in the second part suggests that the content of students’ memory combined with listening and vision will last longer. The audio-visual teaching theory reveals that if students can activate more organs to immerse themselves in learning, their brains can quickly respond and strengthen memory by stimulating “image and sound.” The teaching content should be combined with the actual life of students, so that students can learn and use English vividly without breaking away from the reality, and their interest of learning English can be stimulated.

### Design of Teaching Mode

DL is integrated into the whole teaching process, which fully embodies the creating scene. It is essential to research and analyze the data, explore the dependences lying in factors, and summarize and improve the key links. Primarily, the films are prepared. Four films are selected as the teaching material (“Snow White,” “The Croods,” “The Lion King,” and “Black and white haired witch”), and combined with the course content to be learned, which mainly focus on the present continuous tense. Teaching contents are problem-oriented, and students learn to solve problems through films. Then, teachers, as guides, introduce students randomly into the learning content by presenting pictures, films, audio-visual materials or by other methods. Finally, on the premise of learning and understanding the basic knowledge, students’ thinking ability is cultivated, so that they can obtain their own understanding freely through dialogue, melody and other forms. In the whole learning process, students need to participate in teaching activities, and carry out communication and cooperation between peers and groups, to complete the tasks of situational dialogue, role play and mutual evaluation. Thereby, cooperative learning strategy is also very crucial. In the application of film teaching, teachers design related problems according to the film content in the preparation stage of knowledge teaching, in order to attract students and stimulate students’ interest. Then, the teacher implements the course through the pre class introduction, knowledge transfer, and comprehensive application stage.

### Questionnaire Design

**FIGURE 5:**
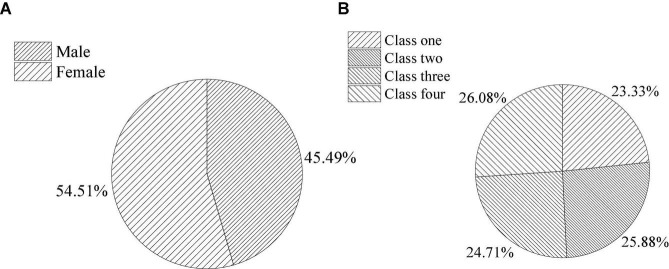
Basic information of the experimenters (**A**, ratio of genders; **B**, ratio of classes).

The film teaching mode is designed for primary school students. There are 300 students selected from four normal classes in the same primary school as the research object. Figure 5 presents the basic information of the experimenters.

The survey lasted for 3 months from October to November in 2020. The questionnaires were pre-investigated, recovered and optimized. In December of 2020, a large-scale analysis and research were carried out. A total of 300 questionnaires were distributed and 272 were recovered, with a recovery rate of 90.66% and 272 valid questionnaires. To verify the reliability, stability and index system of the questionnaire data, the quality of the questionnaire are tested and verified by software on the α reliability coefficient. The design, distribution and data collection of the questionnaires do not infringe any personal privacy. The questionnaires are conducted with the consent of the participants. The questionnaires are approved by the school leaders and relevant departments. The anonymous system is implemented in the questionaires, and the data obtained is only used for academic research. The questionnaires do not involve students’ personal privacy and are approved by the students of the school. The questions included in the questionnaire are described in Table 1.

**TABLE 1:** Key question setting of the questionnaire.

Question distribution	Content and specific options	
Q2	C1: Is it too difficult to learn in the application of film teaching mode in class?	C11 too difficult C12 moderate C13 not difficult
Q4	C2: After the application of film teaching, what is the students’ mastery of knowledge?	C21 Good C22 General C23 Poor
Q6	C3: Do you like film teaching	C31 Like C32 General C33 Dislike
Q8	C4: Is there enough learning resources in video teaching?	C41 Many C42 Appropriate C43 Less
Q10	C5: Do you think the study of English video appreciation class will help you improve your English?	C51 Little help C52 No help C53 Great help C54 Quite great help
Q12	C6: What kind of films do you like?	C61Appreciation of famous works C62 Science fiction film C63 Cartoon
Q13	C7: What kind of form do you want to have English class?	C71 Speaking-based C72 Performance-based C73 Listening-based

### Reliability and Validity Analysis of the Questionnaire

The reliability of the QS is analyzed through SPSS 25.0, and the obtained Cronbach’s alpha is 0.804, which is larger than 0.7 and has good reliability. The results of reliability statistics are presented in Table 2.

**TABLE 2:** Reliability statistics.

Cronbach’s alpha	Standardization item-based Cronbach’s Alpha	Number of items
0.804	0.803	8

The KMO and Barlett’s Spherical tests are conducted using SPSS25.0 on the QS, and factor analysis is carried out on the QS, thus obtaining the validity of the QS. The KMO is 0.657, greater than 0.6, so each question in the QS is suitable for factor analysis and has good constructive validity. The tests of KMO and Bartlett are shown in Table 3.

**TABLE 3:** KMO and Bartlett’s Spherical tests.

Kaiser-Meyer-Olkin for sampling sufficiency	Measurement	0.657
Bartlett’s Spherical Test	Approximate chi-square	143.478
	df	55
	sig	0.000

The above data shows that the questionnaire designed here has good reliability and validity, strong internal consistency and stability, high reliability, reasonable and effective, and can be used as a research tool.

### Analysis of the Results of Student Questionnaire Survey

#### Analysis of Students’ Adaptability

**FIGURE 6:**
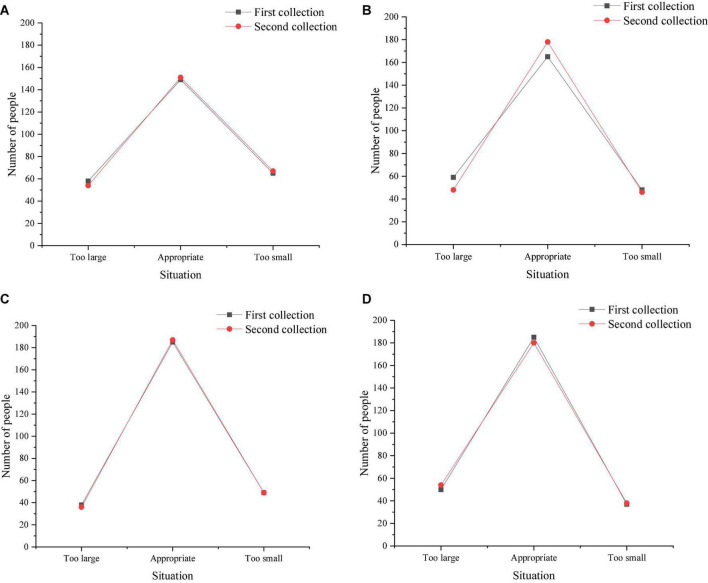
The difficulty of film clips in film teaching (**A**, 2 h; **B**, 4 h; **C**, 6 h; **D**, 8 h).

Figure 6A shows students’ difficulties in adapting the film teaching after the films are played in class for about 2 h. Figure 6B shows students’ difficulties in adapting the film teaching after the films are played in class for about 4 h. Figure 6C shows students’ difficulties in adapting the film teaching after the films are played in class for about 6 h. Figure 6D shows students’ difficulties in adapting the film teaching after the films are played in class for about 8 h.

**FIGURE 7:**
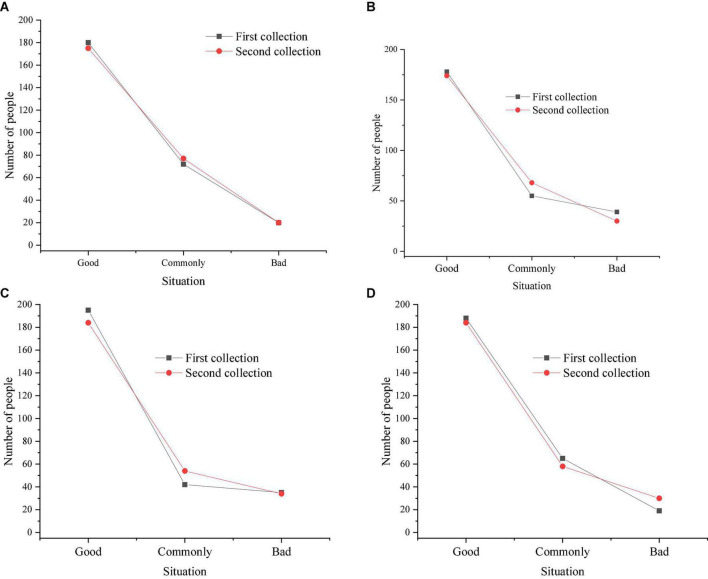
Students’ mastery (**A**, 2 h; **B**, 4 h; **C**, 6 h; **D**, 8 h).

Figure 6 indicates that around 2 h after the beginning of the experiment, about 159 children thought that the video was appropriately difficult, and 68 students thought that some film resources were more than difficult or less than difficult. The reason should be that the film teaching model has just begun to implement, and students are not adaptive for this model. Around 4 h after the beginning of the experiment, about 168 children thought that video was appropriately difficult, and 53 students thought that some film resources were more than difficult or less than difficult. Around 6 h after the beginning of the experiment, about 192 children thought that video was appropriately, and 38 students thought that some film resources were more than difficult or less than difficult. Around 8 h after the beginning of the experiment, about 198 children thought that video was appropriately difficult, and 32 students thought that some film resources were more than difficult or less than difficult. With the continuous growth of the implementation time of film video mode, students gradually adapted to this teaching mode, and the number of students who thought that video was appropriately difficult was increasing. As Figure 6 displays, children’s difficulty in applying movies to the classroom has obvious differences in different test stages. In the test stage with a duration of 2 h, the number of people who think it is more difficult is significantly higher than that in the test stage with a duration of 4 h (*p* > 0.05) and the test stage with a duration of 6 h (*p* > 0.05). There is no significant difference between the 6-h test stage and the 8-h test stage (*p* > 0.05). Figure 7A illustrates students’ mastery of knowledge after around 2 h. Figure 7B indicates students’ mastery of knowledge after around 4 h. Figure 7C signifies students’ mastery of knowledge after around 6 h. Figure 7D refers to students’ mastery of knowledge after around 8 h. After around 2 h of the beginning of the experiment, about 187 students thought that they had a good command of knowledge with the application of film teaching mode, and about 68 students thought that they mastered knowledge appropriately and 17 students thought that knowledge was not well mastered. After around 4 h of the beginning of the experiment, about 197 students thought that they had a good command of knowledge with the application of film teaching mode, and about 58 students thought that they mastered knowledge appropriately and 15 students thought that knowledge was not well mastered. After around 6 h of the beginning of the experiment, about 205 students thought that they had a good command of knowledge with the application of film teaching mode, and about 55 students thought that they mastered knowledge appropriately and 10 students thought that knowledge was not well mastered. After around 8 h of the beginning of the experiment, about 208 students thought that they had a good command of knowledge with the application of film teaching mode, and about 52 students thought that they mastered knowledge appropriately and 12 students thought that knowledge was not well mastered. According to the statistical results, the samples were tested by *t*-test. There was no significant difference among different test duration groups, which are 2-h test group, 4-h test group, 6-h test group and 8-h test group. The children’s knowledge level was basically the same (*p* > 0.05). The possible reason was that the children were young and their knowledge reserve was limited, and the inertial thinking of knowledge has not been formed.

**FIGURE 8:**
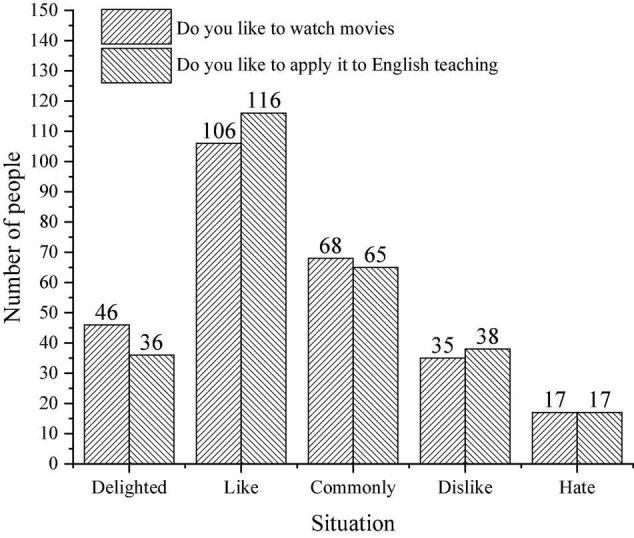
Students’ attitude survey.

The survey of primary school students’ attitude toward using film teaching in English class mainly focuses on two aspects: students like films and students like the application of film teaching in English class. Figure 8 displays the specific situation.

Figure 8 implies that more than half of the students like cinema teaching, and only a few students do not like cinema teaching.

**FIGURE 9:**
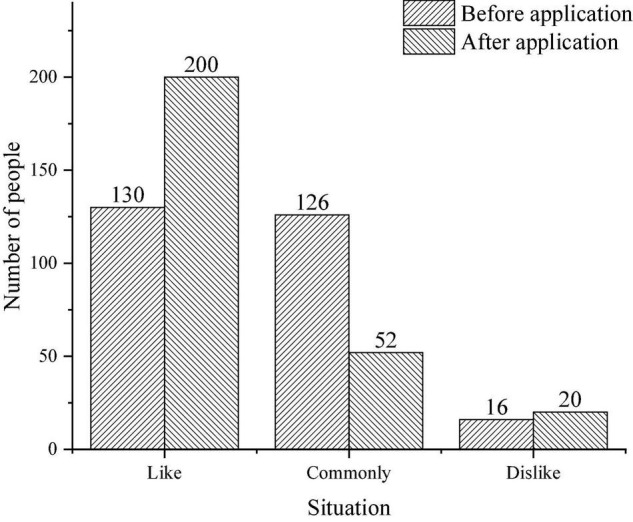
Comparison of students’ attitudes before and after the application of film teaching.

Figure 9 presents the result of questionnaire analysis.

Figure 9 reveals that 126 students have a neutral attitude toward English, and they do not like it or hate it before the application of film teaching. In the evaluation of the English curriculum, more than half of the students are still interested in the English curriculum. Moreover, about half of the students are not interested in the current English course. The application of the teaching film reflects that 130 students are interested in and support the film teaching, and very few of them do not like it.

### Evaluation of Learning Resources

**FIGURE 10:**
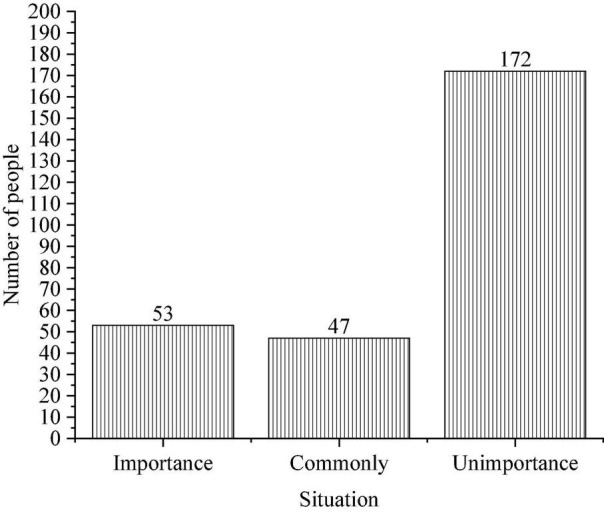
Teacher’s emphasis on western culture.

English learning is not only a process of cultural contact, but also a process of language learning. The content of the film video is very different, and the plot shows the characteristics of knowledge, culture, customs and so on. This kind of knowledge is diversified, which makes students acquire multi-dimensional knowledge in the process of watching films and TV, cultivate cross-cultural communication awareness, and promote future communication and practical application. English is an applied tool. English must be used in practice to be better understood, because it contains rich cultural connotations, representing the western nation. If a student does not understand English culture and history at all, that will inevitably lead to a misunderstanding for the student. There are many similar examples, such as signing a letter in the case of this document. Hence, it is possible to expand students’ view of the world by developing a preliminary understanding of different national cultures, as shown in Figure 10.

Data analysis in Figure 10 illustrates that 172 students think that teachers do not focus on cultural interpretation, and students are more than half likely to encounter cultural barriers in English class. If teachers focus more on cultural interpretation, students will be interested in different national cultures.

**FIGURE 11:**
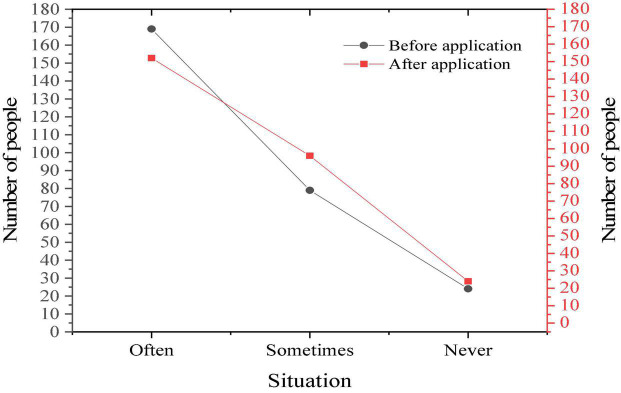
Situation of students before and after using film teaching.

Figure 11 displays the students’ understanding of western culture through film teaching.

As Figure 11 suggests, most students think that film teaching is conducive to the understanding of western culture, so they are more interested in English.

### Analysis of Student Interview Results

Table 4 reveals the student interview record.

**TABLE 4:** Questions related to student interviews.

**Interview questions**
Q1 Do you like the way of film teaching?
Q2 What do you think are the advantages of film teaching?
Q3 Do you adapt to the way of film teaching?
Q4 What difficulties do you think film teaching will encounter in class?

Students are randomly selected and interviewed to express their opinions as accurately as possible. During the whole interview, the questions are random. The analysis of the interview results shows that a considerable number of students are willing to participate in the teaching and hold a positive attitude toward the application of film and video teaching in classroom English teaching. The classroom atmosphere is very good, the enthusiasm degree is relatively high, and the knowledge memory is very deep, which can show their team and cooperative game situation, thus increasing the communication between students and teachers, determining the correct values of the story of the film, and publicizing the western elements.

However, the implementation of film teaching also brings some problems to some students. The speed of the film is too fast to understand, and the students are unwilling to speak out. There are some difficult factors in the learning process, such as the attitude toward the film video teaching mode. Of course, these results are just a random interview results, while students’ specific views and attitudes on the application of teaching mode in English class on film and TV cannot be sure. They are only explored through the questionnaire.

### Analysis of Teachers’ Feedback

Table 5 is the interview record from teacher.

**TABLE 5:** Questions asked to teacher in the interview.

**Interview questions**
Q1 What new teaching methods does the teacher try in the process of teaching?
Q2 What difficulties will teachers encounter in the teaching process?
Q3 What are the changes in the application of film teaching?
Q4 What do teachers think can be improved in film teaching?

Interviews on teacher have been studied. Teachers’ views on the application of film teaching in primary school English teaching are analyzed. The implementation of the film and video teaching in English teaching of primary school can provide a broader platform for students. Traditional education includes oral and audio-visual resources, film and video sound, image and text. Most students are willing to actively participate in these activities. The cooperation with many specific activities can improve students’ communication ability and cooperation consciousness, so that students can make full use of what they have learned and cultivate their interest in learning multicultural knowledge. However, things are usually two-way, and film teaching also have shortcomings. Teachers say that if every lesson is presented in this way, the workload of teachers will increase at the beginning of teaching. In addition to a certain demand for teachers’ information technology skills, teachers also need massive film resources to choose the appropriate part to combine the teaching content. In the classroom environment, teachers should design appropriate teaching activities, and correctly and timely guide and explain doubts to students, which will lead to classroom confusion and make classroom discipline difficult to control. Most homework after class needs multi-link evaluation, and students’ presentation content also needs to be evaluated from multiple perspectives, which is different from the traditional single evaluation and also increases the content of the teacher’s work.

Compared with other methods, the advantage of the method proposed here is to focus on the unique teaching form of English films clips that can attract primary school attention, and make full use of its advantages in classroom atmosphere and language teaching. What is studied and discussed is the positive role of English films clips in primary school English classroom teaching. Compared with other schemes proposed by other people, the scheme proposed here introduces deep learning and multicultural learning to attract students’ interest in learning.

Table 6 summarizes the research achievements and findings.

**TABLE 6:** Summarization of the research achievements and findings.

Foundings and results in “Positive psychology and film teaching theory” Section	Foundings and results in “Analysis of the results of student questionnaire survey” Section	Summarization
Children can better memorize information obtained through various channels.	With the increasing duration of carrying out the film teaching mode, there is an increasing number of children adapting to film teaching mode. The difficulty of selected teaching materials should be moderate.	1. The film teaching mode improves children’s learning interest and learning motivation. The English film teaching provides a virtual language environment and cultural environment. With the help of pictures and sounds, it can make the classroom vivid and stimulate students’ learning interest. Students are also more actively involved in teaching activities. The stimulation of intrinsic motivation is also more conducive to the progress of students’ language learning. 2. The film teaching mode improves children’s language skills. Under the designed scenes of film teaching, students can perceive functions of English language in the context. The comprehensible language promotes students’ listening ability and oral expressing ability of English language. Children are attracted by the pictures and images, where movie characters’ body language and even voice intonation can help them better absorb knowledge from the film and enhance their understanding of the content. 3. The film teaching mode fosters children’s western cultural literacy. Students like to learn western customs and values in a relaxed environment. Movies can intuitively show the humanistic style, historical geography, customs and so on of English countries, because the culture close to daily life can arouse learners’ resonance.

## Discussion and Comparison

By comparing the methods proposed here with those proposed by Gajdo and Korpa (2019), they focussed on how to teach grammar by using scripts and fragments of the legendary movie “Revenger Alliance“. And, comparing the methods proposed here with those proposed by Bunting et al. (2020), they focussed on the prospective system of children’s personalized reading skills development in English classes, who believed that personalized learning technology was a promising method to alleviate differences in children’s reading abilities. Different from the former, advantages of the methods proposed here lies in starting from the anasis on the current situation of children’s English learning, introducing movies to aid English teaching, which are selected, edited and elaborated according to the teaching contents, and implementing teaching work, which integrates “watching, listening. speaking, reading and writing” and achieves great teaching effect. Nowadays, parents pay gradually more attention to children’s bilingual education, which has become a trend today. It is beneficial for children developing their language ability in the future to have access to English during the developing stage of their language ability so that children can express themselves in both Chinese and English.

## Conclusion

Primary school English teaching should be student-centered, and teaches should not only impart knowledge, but also guide students to think more. Results show that the form of children’s English teaching is single, and English movies can attract children’s attention, whose advantages are fully used. And it is adopted in primary school English teaching class. The experimental results show that the teaching strategy of films segment can not only solve some problems in current English teaching, but also improve the quality of classroom English teaching and lay the foundation for the sustainable development of students. Multiculture is an important foundation for the study of teachers’ teaching efficiency in colleges and universities. Some references are provided for the implementation of English movies in children’s English teaching.

There are also some aspects to be improved. First of all, there is certain limitation of the number of the surveyed school, which is due to the limitation of time and energy. The same question also exists in the selection of the research samples. Second, study is only conducted on the close relationship between learning theories under the Constructivism and English movies, which does not integrate information technology into English film teaching or deeply explore the construction of English film resource library and English learning center. The research on English film teaching is in the preliminary stage, with much research space and great practical value. It is hoped that more researchers will conduct deeper research and continuous reflection.

## Data Availability Statement

The raw data supporting the conclusions of this article will be made available by the authors, without undue reservation.

## Ethics Statement

The studies involving human participants were reviewed and approved by the Guangxi Medical University Ethics Committee. The patients/participants provided their written informed consent to participate in this study. Written informed consent was obtained from the individual(s) for the publication of any potentially identifiable images or data included in this article.

## Author Contributions

All authors listed have made a substantial, direct, and intellectual contribution to the work, and approved it for publication.

## Conflict of Interest

The authors declare that the research was conducted in the absence of any commercial or financial relationships that could be construed as a potential conflict of interest.

## Publisher’s Note

All claims expressed in this article are solely those of the authors and do not necessarily represent those of their affiliated organizations, or those of the publisher, the editors and the reviewers. Any product that may be evaluated in this article, or claim that may be made by its manufacturer, is not guaranteed or endorsed by the publisher.
